# Plant Biomimetic Principles of Multifunctional Soft Composite Development: A Synergistic Approach Enabling Shape Morphing and Mechanical Robustness

**DOI:** 10.1021/acsbiomaterials.3c01163

**Published:** 2024-02-21

**Authors:** Gital Shteinberg, Rami Haj-Ali, Flavia Libonati, Mirit Sharabi

**Affiliations:** † Department of Mechanical Engineering and Mechatronics, Ariel University, Ariel 407000, Israel; ‡ School of Mechanical Engineering, Tel Aviv University, Tel Aviv 6997801, Israel; § Department of Mechanical, Energy, Management and Transportation Engineering, University of Genoa, Genova 16145, Italy

**Keywords:** materials design, shape transformation, soft Composites, mechanical behavior, bioinspiration, hydrogels

## Abstract

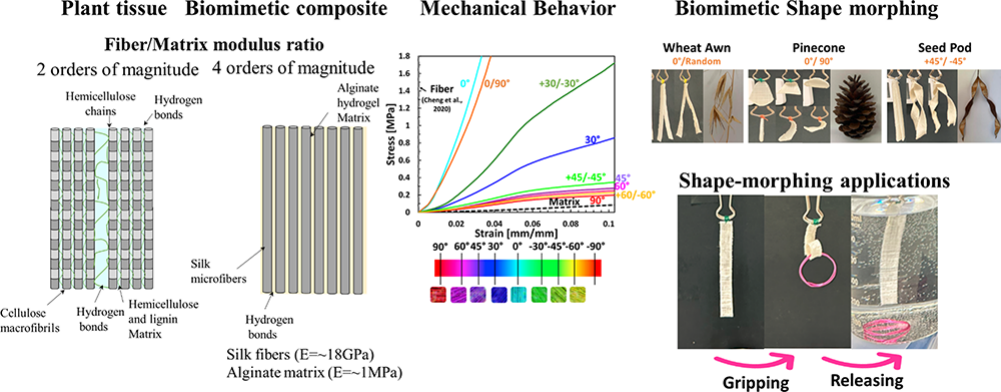

Plant tissues are constructed as composite material systems of stiff cellulose microfibers reinforcing a soft matrix. Thus, they comprise smart and multifunctional structures that can change shape in response to external stimuli due to asymmetrical fiber alignment and possess robust mechanical properties. Herein, we demonstrate the biomimetics of the plant material system using silk fiber-reinforced alginate hydrogel matrix biocomposites. We fabricate single and bilamellar biocomposites with different fiber orientations. The mechanical behavior of the biocomposites is nonlinear, with large deformations, as in plant tissues. In general, the bilamellar system shows increased modulus, strain UTS, and toughness compared to the single-lamellar system for most of the tested orientations. Overall, the biocomposites present a wide range of elastic modulus values (3.0 ± 0.6–104.7 ± 11.3 MPa) and UTS values (0.23 ± 0.04–12.5 ± 2.0 MPa). The bilamellar biocomposites demonstrated shape-transforming abilities with diverse morphing modes, emulating different plant tissues and creating complex shape-morphing structures. These multifunctional biocomposites possess tunable and robust mechanical properties, controllable shape-morphing deformations, and the ability to self-controlled encapsulation, grip, and release objects. By harnessing biomimetic principles, these soft, smart, and multifunctional materials hold potential applications spanning from soft robotics, medicine, and tissue engineering to sensing and drug delivery.

## 1 Introduction

In recent years, mechanically active, self-shaping materials have gained significant interest for their ability to transform three-dimensional (3D) shapes on demand when triggered by external forces. This fantastic ability, inspired by the billion-year-long plant evolution, allowed nature to design both simple and complex structures that display diverse shapes and functions. These structures can change shape in response to external stimuli.^1,2^ For example, in response to an electrical stimulation that releases turgor pressure, the *Venus flytrap* firmly clamps its leaves on the insect in a split second.^1,3,4^ The *Mimosa pudica* quickly folds its leaves downward when exposed to wind, vibration, or touch. This reaction serves as a defense mechanism, offering protection against animals and certain insects.^4,5^ A biochemical process of the action potential is responsible for the fast movements in which motor cells lose their turgor pressure.^5,6^ The seedpods, wheat awns, and pinecones release their seeds by twisting and bending deformations in response to humidity changes in the air.^3,7,8^

Plant tissues are multiscale soft composites of aligned stiff cellulose microfibrils embedded in a soft hygroscopic matrix composed mainly of hemicellulose, pectin, and lignin.^3,9,10^ This remarkable structure allows the plant to control its shape, size, mechanical properties, and specific movements.^11^ Plant tissues change their shape primarily due to an asymmetrical fiber alignment, resulting in a coupling effect between their in-plane, bending, and twisting deformations.^12^ The creation of bioinspired material systems that can morph in a controlled manner, as seen in nature, is paramount in many fields of fundamental and applied sciences. From biomedical devices to aircraft design, self-shaping materials are of great interest because of their wide range of applications.^13^ Hydrogels, composites, polymers, and tissues may all alter form in response to external stimuli.^14−16^ Diverse fabrication techniques such as patterning, molding, microfluidics, electrospinning, and 4D printing are used,^17^ and various factors affect the final shape, such as material dimensions,^18−20^ the patterned structure,^17^ mechanical properties of the components,^21^ fiber orientation,^1,7,22,23^ and the different stimuli.^2,17^

Nevertheless, hydrogels have the highest potential as a shape-morphing material due to drastic volumetric expansion or contraction abilities because of the influx and efflux of water that creates internal stresses, which induce bending, twisting, stretching, buckling, or wrinkling deformations.^15,24,25^ Moreover, hydrogels and hygroscopic materials are superior due to their stimuli-responsiveness to various triggers such as humidity, temperature, electric or magnetic field, light, pH, etc.^26,27^ However, their mechanical properties are mostly inferior. Recently, shape-morphing hydrogels with robust properties such as increased toughness and strength were developed.^28−31^

While various hydrogels are being engineered with morphing capabilities, and separately, different hydrogels are being developed to exhibit enhanced stiffness, strength, and toughness (often focusing on one property at a time), the occurrence of new materials that combine both characteristics within a uniform material remains rare. However, within biological materials like plants, this combination is inherent. Unlike synthetic materials, soft biological materials, designed as fiber-reinforced hydrogel composites, exhibit robust mechanical properties alongside additional functionalities, such as morphing abilities. Consequently, diverse plants can alter their shapes while maintaining structural integrity and mechanical robustness.^32^ These materials are based on relatively simple building blocks, diverse structural motifs, and multiscale hierarchy.

In this work, we present a multifunctional biocomposite material with robust mechanical properties and shape transformation abilities based on plant tissue biomimetic principles. The material is based on natural silk fiber-reinforced biocomposite with single- and bilamellar structures arranged at different fiber orientations (0°, 30°, 45°, 60°, and 90°). Both the single and bilamellar composites demonstrate anisotropic nonlinear behavior with large deformations. Moreover, they exhibited tunable stiffness, strength, and toughness controlled by modifying the fiber orientation. Here, we demonstrate that the bilamellar composites can achieve improved mechanical properties compared with the single-lamellar composites for similar fiber orientations. Furthermore, our bilamellar biocomposite shows programmable, controllable, and reversible shape-morphing abilities: from 2D planar structures into various controllable 3D structures, including helixes, tubes, rolls, and additional complex structures and can be used for gripping and programmable releasing of objects.

## 2 Experimental Section

### 2.1 Fiber Preparation

Silk cocoons (*Bombyx mori*) are immersed in boiling double-distilled water (DDW) at 100 °C for 30 min to soften the cocoons. The boiled cocoons are cooled off to room temperature and then rinsed three times with DDW. The soft silk cocoons are stored in fresh DDW at room temperature. The silk fibers are easily extracted and manually separated from the cocoon by gently pulling them with tweezers. The separated fibers are manually wrapped around thin 3D-printed frames to fabricate silk laminates with different fiber orientations.

### 2.2 Fiber Orientation

The extracted silk fibers are manually wrapped around thin 3D-printed frames in different quadrangular shapes to produce an array of fibers in a specific orientation. The aligned fibers are formed in five orientations with the vertical axis, 0° (longitudinal), 30°, 45°, 60°, and 90° (transverse). The fiber orientation is controlled by the frame configuration design. To produce fiber frames at an angle of 0° or 90° 3D-printed square frames (33 × 33 × 1 mm) or rectangular frames (14 × 95 × 1 mm) are used. For fiber orientations of 30°, 45°, and 60°, parallelogram frames are designed (28 × 119 × 1, 19.8 × 109 × 1, and 16 × 103 × 1 mm, respectively), where the frame’s angle defines the fiber wrapping direction. Single-lamellar and bilamellar laminates are fabricated. The single-lamellar biocomposite is constructed from a single fiber framed into a specific orientation (0°, 30°, 45°, 60°, or 90°). The bilamellar biocomposite is designed with two inverted fiber frames, which are face-to-face assembled to create various combinations, such as cross-plied laminate (0°/90°) and angle-plied laminates (+30°/–30°, +45°/–45°, and +60°/–60°).

### 2.3 Biocomposite Laminate Fabrication

**Figure 1:**
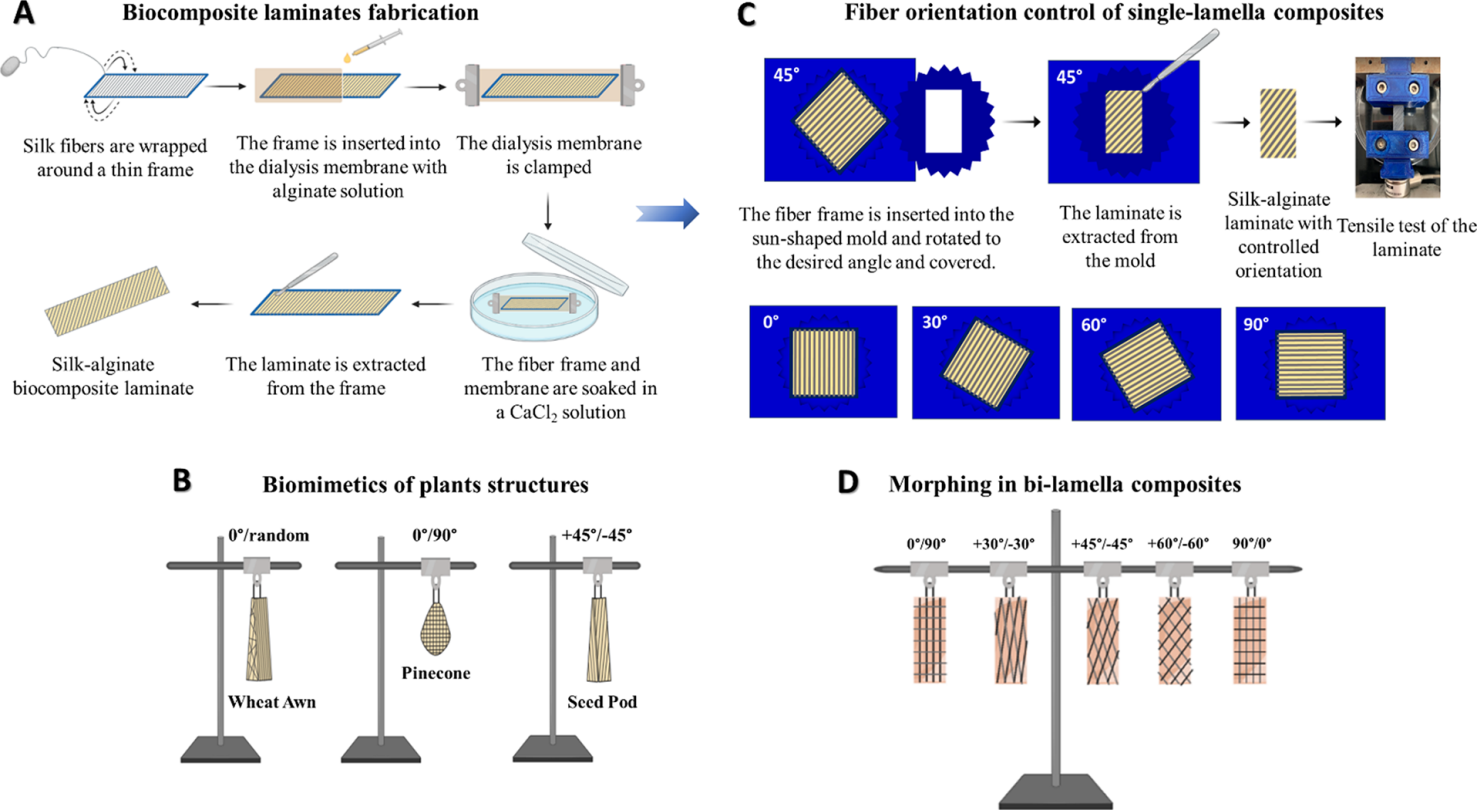
Illustration of the fabrication, mechanical characterization, and morphing experiments. (A) Laminates biocomposite fabrication. Silk fibers are wrapped around thin custom 3D-printed frames. The fiber frame is inserted into dialysis cellulose membranes with a sodium alginate solution. The dialysis membranes are clamped on both sides using clips and inserted into a CaCl_2_ solution to cross-link the alginate. The laminates are extracted from the frames using a surgical knife and cut into several samples. (B) Biomimetics of plant structures. The wheat awn is made of a two-layer laminate of 0°- and random-fiber orientations. The pinecone is constructed of cross-plied 0°/90° laminate. The seedpod is composed of two +45°/–45° bilamellar composites. (C) Fiber orientation control of single-lamellar composites. Fiber orientation-controlled pattern for fabricating the biocomposite laminates using sun-pattern for tensile testing. A custom 3D-printed sun pattern is designed to control the fiber orientation of the biocomposite laminates during cutting. Tensile tests are performed on the resulting biocomposite laminates. (D) Morphing in bilamellar composites. Bilamellar biocomposites are hung at room temperature vertically until completely dried.

The biocomposites are fabricated as previously described.^33,34^ Silk fibers are manually wrapped around thin 3D-printed frames to create a unidirectional and organized array of fibers. The fiber thickness on each frame is measured using a digital micrometer at ten locations at least. The frames with the oriented silk fibers are inserted into a dialysis tubing cellulose membrane (MWCO 14000, Sigma-Aldrich, Israel). For single-lamella, only one fiber frame in a specific orientation is inserted. For bilamella, two fiber frames are inserted into the dialysis membrane, placing one on the other. The membrane is filled with a 6% (w/v) sodium alginate (Protonal LF 10–60, FMC biopolymer, USA) solution in DDW. The alginate solution is spread uniformly on both sides of the fiber frame and then manually flattened to fill all the empty spaces between the fibers, to prevent the formation of air bubbles. Subsequently, the dialysis membrane is sealed on both sides and then soaked in 0.1 M CaCl_2_ in DDW (Sigma-Aldrich, Israel) for at least 48 h at room temperature to cross-link the alginate hydrogel. The calcium ions diffuse into the membrane, creating hydrogen bridges with the alginate and generating a hydrogel matrix between the fibers. Finally, the laminates are extracted from the frames using a surgical knife and kept in the CaCl_2_ solution until the mechanical tests and morphing experiments. The fabrication process is described schematically in Figure 1A.

### 2.4 Biomimetics of Plant Architectures and Complex Structures

Bilamellar biocomposites are used to mimic the morphing behavior of various plants, such as seedpods, wheat awns, and pinecones (Figure 1B). The seedpod structure is constructed from two bilamellar +45°/–45° biocomposites assembled inversely face to face.^19,27^ The wheat awn architecture is composed of two identical inverted bilamellar biocomposite strips assembled face to face. Each ribbon is fabricated from two fiber frames: the first is oriented at 0° while the second is randomly oriented.^7,11^ The pinecone scales are cut using a 3D printed scale-shaped mold from cross-plied 0°/90° laminates. These orientations are based on the synthetic analog of the pinecone scales presented by Studart and Erb.^1^ In particular, cellulose microfibrils in the upper layer of the scale are aligned along the length of the scale and the lower layer consists of sclereids with predominant cellulose direction perpendicular to the plane of the scale. Self-designed complex structures from diverse bilamellar laminates are cut from the prepared laminates according to dedicated 3D-printed molds.

### 2.5 Fiber Quantification and Orientation Characterization

The fiber volume fraction (FVF) is defined as the thickness ratio calculated by dividing the fiber thickness by the biocomposite laminate thickness. The oriented silk fibers are photographed on a black background (Jiusion digital microscope), and the images are processed using ImageJ (NIH) software with an OrientationJ plugin^35^ to capture the fiber orientation.

### 2.6 Fiber Orientation Control of Single-Lamellar Biocomposites

A custom-made 3D-printed angle-controlled sun-shaped mold is used to cut the laminate samples in the required fiber orientation of 0°, 30°, 45°, 60°, or 90°. The sun-shaped mold is designed as a thin rectangular plate with a sun-shaped hole with 24 rays, where each ray has a shifting angle of 15°. Additionally, a custom-made 3D cover of sun-shaped with 24 rays with a rectangular hole is designed to allow refined cutting of the laminates. A square frame of a single-lamellar biocomposite is inserted into the sun-shaped mold, and by rotating it, the desired orientation is obtained. Turning each time in one ray adds an angle of 15° to the fiber orientation. When the desired angle is chosen, the sun-shaped cover is placed on, and the samples are extracted from the rectangular hole using a surgical knife, as shown schematically in Figure 1C. The cut samples are then tested mechanically.

### 2.7 Mechanical Testing

The single-lamellar and bilamellar biocomposites are tested under uniaxial tension. The single-lamellar biocomposites are tested at five orientations (i.e., 0°, 30°, 45°, 60°, and 90°) with respect to the longitudinal axis (the tensile direction); the bilamellar biocomposites are tested at cross-plied orientation (0°/90°) and three angle-plied orientations of +30°/–30°, +45°/–45°, and +60°/–60°, with respect to the tensile direction. Tensile testing is performed using a tensile machine endowed with a 222 or 22 N load cell (Psylotech μTS system, IL, USA) under a quasi-static displacement rate of 3 mm/min using a displacement control mode. According to the tensile protocol, samples of 0°, 30°, 0°/90°, and +30°/–30° are initially prestretched manually until 2 N using the 222 N load cell. At the same time, other orientations started from a zero-load position of 0 N with the 22 N load cell. For stiffer samples, preloading was performed. Then all samples are proceeded by five preconditioning cycles at a constant rate of 3 mm/min to 5% strain of their original length. After the preconditioning phase, the samples are stretched to failure. The width and thickness are measured at three different locations along the sample using a digital caliper and a micrometer, respectively. The average geometric dimensions are used to calculate the cross-sectional area and FVF. The FVF is defined as a ratio between fiber thickness and laminate thickness. The laminate samples are gripped in the tensile machine using custom-3D printed clamps, and the gage length of each sample is measured as the distance between the clamps. The aspect ratio of the sample is calculated as the width-to-length ratio. All samples are kept in the CaCl_2_ solution until the tensile test.

### 2.8 Mechanical Behavior Characterization

The tensile behavior of silk biocomposites is analyzed as a function of fiber orientation, and the mechanical properties are calculated as engineering stresses and strains. The stress and strain are calculated as the ratio of the force to the initial sample’s cross-sectional area and as the elongation difference of the sample divided by the original length, respectively. The mechanical behavior is described as a stress–strain curve. The maximum stress before failure is expressed as the ultimate tensile strength (UTS), and the ultimate tensile strain is defined as the strain at UTS. The elastic modulus is determined as the slope of the initial linear section of the stress–strain curves (between 1.5 and 6.5% strain) for all samples. The linearity is confirmed by the *R*^2^ value between 0.9975 and 1. Toughness is defined as the area under the stress–strain curve and calculated up to the failure point using the trapezoidal rule. At least five tensile tests are performed for each group of manufactured laminates.

### 2.9 Morphing in Bilamellar Composites

Bilamellar composites (0°/90°, +30°/–30°, +45°/–45°, and +60°/–60°) are tested for their ability to transform their shape during dehydration at room temperature. Parallelogram frames of 30°, 45°, and 60° and rectangular frames of 0° and 90° are used to fabricate long bilamellar strips. The average geometric measurements are used to calculate the aspect ratio and FVF. The bilamellar samples are hung lengthwise on a rod stand and tied for stabilization (Figure 1D). Since boundary conditions could affect the shape transformation, the samples are not held directly through the clips. The bilamellar ribbons are hung vertically at room temperature (25.0–26.2 °C) with a relative humidity of 41–46% for approximately 7 h. The laminates are photographed to capture the shape transformation during dehydration until completely dry. The orientation of hanging bilamella is defined by two angles: the fiber angle of the forward layer and the fiber angle of the backward layer. At angles of 30°, 45°, and 60°, there is an effect on the directionality of the fibers with the longitudinal direction. A positive fiber orientation is expressed as an angle that creates a positive slope with the longitudinal axis. For bilamellar +45°/–45° laminate strips, the width factor on the final configuration is examined by comparing small width (6.5 mm) vs large width (8.8 mm), while the length of the samples is kept constant.

### 2.10 Morphing Characterization

The diameter, pitch, and number of turns are analyzed as a function of fiber orientation to determine the final chiral morphology of morphed bilamellar composites. The pitch is defined as the distance between the centers of two successive helix turns. The parameters of diameter and pitch are measured using ImageJ software with the ROI Manager function analysis tool.

Moreover, for each sample, a shrinkage ratio is defined and calculated as moisture content after dehydration, according to eq 1.
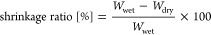
1Here, *W*_wet_ represents the weight of the swollen wet sample before dehydration, and *W*_dry_ represents the weight of the dried sample.

### 2.11 Morphing Reversibility

The reversible process of morphing is defined as the ability of the bilamellar biocomposite to return to its initial shape after dehydration (shrinkage) or hydration (swelling). The reversible ability from shrinkage to swelling and again to shrinkage is examined for +45°/–45° and +60°/–60° bilamellar composites. The reversibility is examined after an initial process, including partial dehydration of the laminates for approximately 3 h and then their immersion in 1 M NaCl solution (hydration process) while being hung vertically. After returning to their original planar configuration, they are removed from the solution and again undergo dehydration until completely dry. For dehydration, the shrinkage ratio is calculated, while the hydration process is defined as a swelling ratio that expresses the absorbed water content according to eq 2.
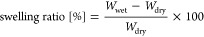
2

### 2.12 Statistical Analysis

Mean, variance, and standard deviation (SD) are obtained for all measurements. Comparisons between laminate groups with similar fiber orientations are made using GraphPad software (GraphPad Prism version 9.4.1, CA, USA). The statistical analysis of the data is done by standard one-way ANOVA test with multiple comparisons. A value of *p* < 0.05 is considered statistically significant.

## 3 Results

**Figure 2:**
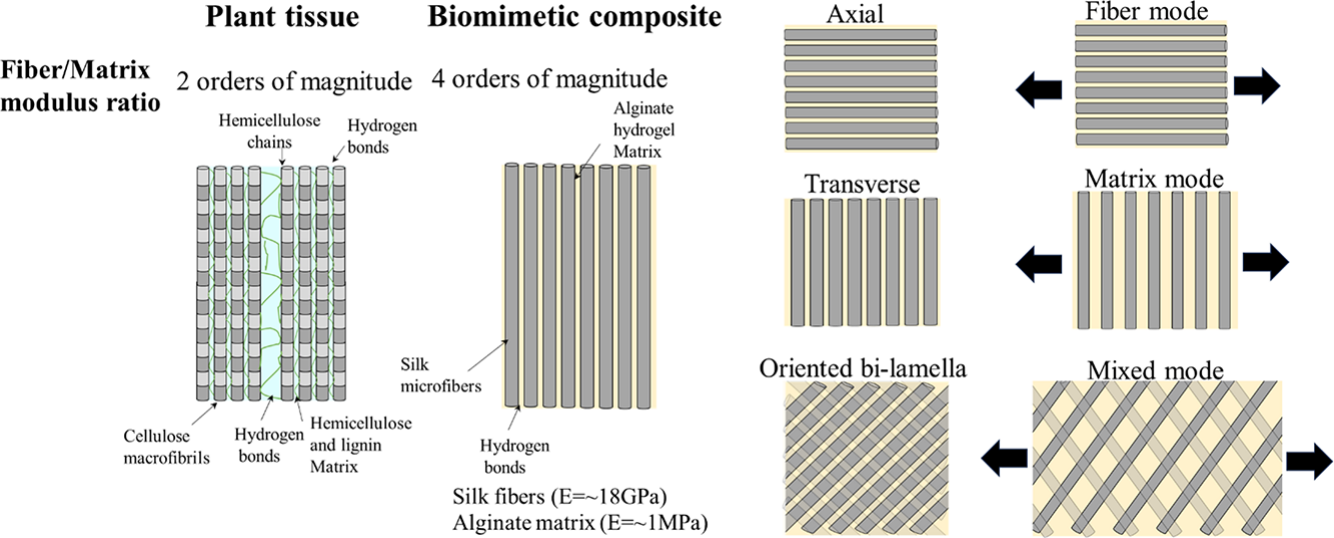
Biomimetics of plant tissues using silk-alginate soft composites using hydrophilic components, an interface of hydrogen bonds, and the extreme difference between fiber/matrix moduli. The fiber orientation governs the mechanical behavior and allows controllable mechanical behavior.

The structure of plant-based composites allows robust mechanical properties with shape-morphing ability due to the considerable stiffness difference between the fibers (cellulose) and polysaccharide (Hemicellulose and Lignin) matrix mediated by the weak interface of hydrogen bonds (2 orders of magnitude).^11^ Our biomimetic composite includes stiff silk fibers (*E* = ∼18 GPa)^36^ embedded in a polysaccharide alginate hydrogel matrix (*E* = ∼1 MPa) (4 orders of magnitude). Under uniaxial tension, the mechanical behavior of our axial soft composites is governed by the fibers, while the matrix mode rules the transverse composites. In large angles, the oriented bilamellar composites are governed by the mixed mode and thus demonstrate an increased ability to deform, as in plant tissues (Figure 2).

Single and bilamellar silk composites are fabricated from silk fibers embedded in an alginate hydrogel matrix in different orientations. The analysis of the orientation distribution confirms the consistency and uniformity of the fiber orientation. A very narrow scatter and small variation around the desired angle are observed for all the tested bilamellar samples 0°/90°, +30°/–30°, +45°/–45°, and +60°/–60°, as shown in Figure S1. This demonstrates the accuracy of the protocol and the ability to produce the laminates in varied orientations. The geometric dimensions and mechanical properties of all tested samples are detailed in supplementary Table S1. The geometric measurements, FVF, and aspect ratio are kept similar to reduce their influence on the mechanical characterization and to allow comparison within each group.

**Figure 3:**
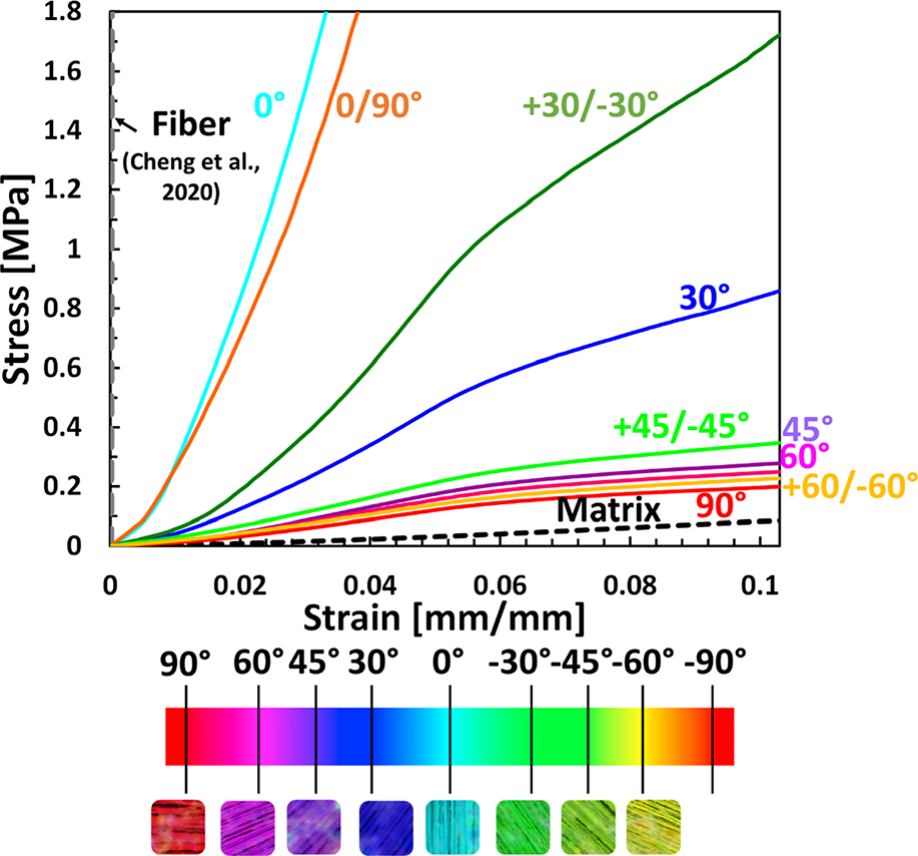
Mechanical behavior of single and bilamellar biocomposites, including Fiber orientation (single lamellar: 0°, 30°, 45°, 60°, and 90° and bilamellar: 0/90°, ±30°, ±45°, and ±60°). The mechanical behavior of the silk fiber^36^ and alginate matrix is also presented as a stress–strain curve. *n* = 6 for 0°, 30°, 45°, 60°, 90°, 0/90°, and *n* = 5 for ±30°, ±45°, and ±60°.

**Figure 4:**
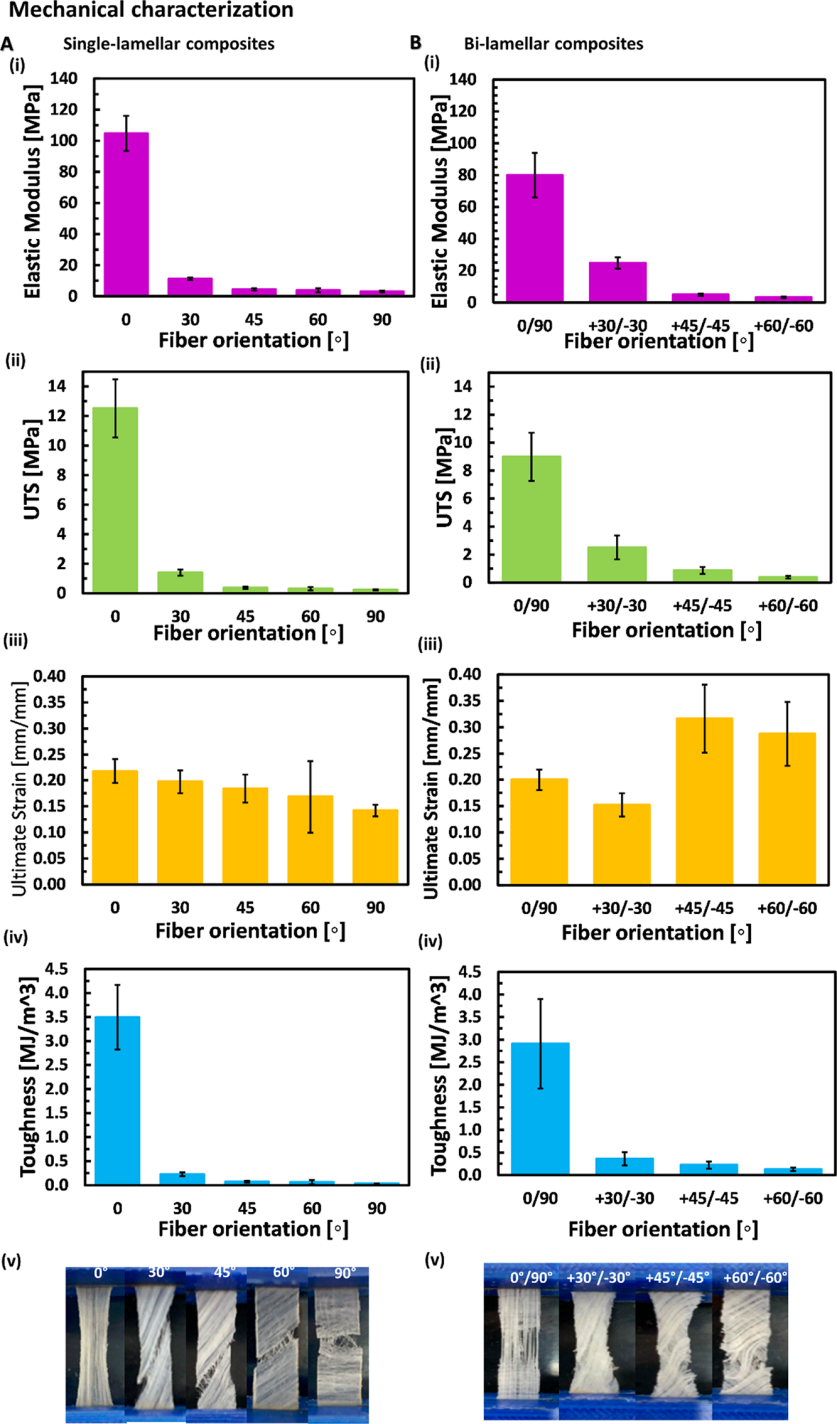
Mechanical characterization of single (A) and bilamellar biocomposites (B). (i) Elasticity modulus, (ii) UTS (ultimate tensile strength), (iii) ultimate tensile strain, (iv) toughness, and (v) failure. *n* = 6 for 0°, 30°, 45°, 60°, 90°, 0/90°, and *n* = 5 for ±30°, ±45°, and ±60°.

Figure 3 shows the mechanical behavior of the tested single-lamellar and bilamellar biocomposites as a function of the fiber orientation (single-lamellar: 0°, 30°, 45°, 60°, and 90° and bilamellar: 0/90°, ±30°, ±45°, and ±60°) and the fiber orientation of the laminates as a color map. The general behavior for all the examined laminates is nonlinear, with large deformations. Figure 4 demonstrates the mechanical properties of the single and bilamellar laminates. For the single lamellar laminates, decreased fiber angle from 90° to 0° results in a steeper curve gradient and higher laminate stiffness Figure 4A(i)). Figure 4A(ii–iv) shows the trends obtained for each mechanical property of single-lamellar biocomposites: increased fiber angle (from 0°, 30°, 45°, 60° to 90°) results in a decrease in elastic modulus, toughness, UTS, and ultimate strain. The elastic modulus, UTS, and toughness show a drastic decline between 0° and 30°-oriented laminates and then a more moderate decrease between 45°, 60°, and 90°-oriented laminates. The stiffest single-lamellar laminate was 0°, then in descending order, 30°, 45°, 60°, and 90°, which is the least stiff. The largest mechanical properties are observed for 0° laminate with 104.7 ± 11.3 MPa, 12.5 ± 2.0 MPa, and 3.5 ± 0.7 MJ/m^3^ for the elastic modulus, UTS, and toughness, respectively. For the 30° laminate, the elastic modulus, UTS, and toughness are 11.2 ± 0.7 MPa, 1.4 ± 0.2 MPa, and 0.23 ± 0.04 MJ/m^3^, respectively. The single-lamellar laminate failure occurs in the matrix along the fiber orientation (Figure 4A(v)).

The differences between the 45° and 60°-oriented laminates are minor in terms of elastic modulus (4.4 ± 0.7 and 3.8 ± 1.4 MPa, respectively) and UTS (0.37 ± 0.07 and 0.31 ± 0.12 MPa, respectively). The lowest elastic modulus (3.0 ± 0.6 MPa) and UTS (0.23 ± 0.04 MPa) are observed for the 90° laminate, which is the least stiff. The lowest toughness values of 0.07 ± 0.02, 0.06 ± 0.05, and 0.03 ± 0.00 MJ/m^3^ are obtained for 45°, 60°, and 90°-oriented laminates, respectively. The ultimate strain values are of the same order of magnitude; the largest strain (0.22 ± 0.02 mm/mm) is observed for 0°-oriented laminate and the lowest strain (0.14 ± 0.01 mm/mm) for 90°-oriented laminate.

In the bilamellar laminates, as in the single-lamellar samples, increased fiber angle from 0°/90°, +30°/–30°, +45°/–45° to +60°/–60°, results in smaller stiffness and strength (Figure 4B(i)). The elastic modulus, UTS, and toughness decrease with the increased angle. This decline is steeper for the first two orientations (Figure 4B(ii–iv)). In contrast, the ultimate strain does not present a similar trend with increased angles (Figure 4B(iv)). The most considerable ultimate strain (0.32 ± 0.06 mm/mm) is observed for +45°/–45° bilamellar samples, whereas the lowest strain (0.15 ± 0.02 mm/mm) is observed for the +30°/–30° laminate. The 0°/90° laminate is the stiffest, with the greatest elastic modulus, UTS, and toughness (79.9 ± 14.0 MPa, 9.0 ± 1.7 MPa, and 2.9 ± 1.0 MJ/m^3^, respectively). The least stiff and strong is the +60°/–60° laminate, with the lowest elastic modulus, UTS, and toughness values (3.2 ± 0.4 MPa, 0.38 ± 0.09 MPa, and 0.13 ± 0.04 MJ/m^3^, respectively). Figure 4B(v) presents the failure of bilamellar laminates by fiber angle combinations. The failure in the 0°/90° laminate is mostly seen in torn 90°-oriented fibers and not in the 0°-oriented fibers.

**Figure 5:**
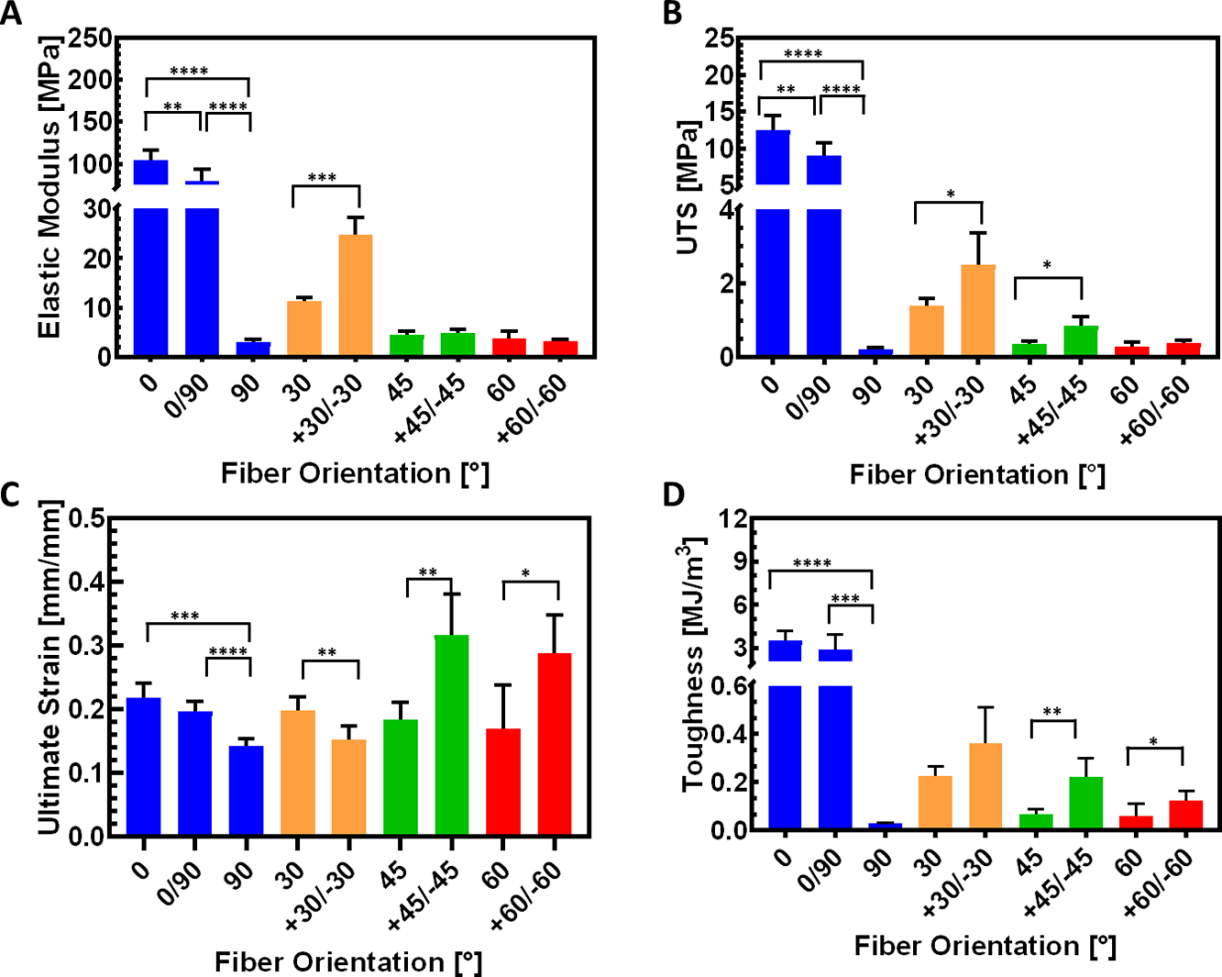
Comparison between single-lamellar and bilamellar laminates: the mechanical properties as a function of fiber orientation. (A) Elastic modulus, (B) UTS, (C) ultimate tensile strain, and (D) toughness. Data are presented as mean ± SD (*p < 0.05, **p < 0.005, ***p < 0.0005, ****p < 0.0001). *n* = 6 for 0°, 30°, 45°, 60°, 90°, 0/90°, and *n* = 5 for ±30°, ±45°, and ±60°.

Figure 5 compares the mechanical properties of the single and bilamellar composites with similar angle combinations. The elastic modulus of 0°/90° laminate is 24% lower than 0° laminate (*p* < 0.005) but 2560% greater than that of 90°-oriented laminate (*p* < 0.0001). The bilamellar +30°/–30° samples demonstrate a significantly larger elastic modulus than the single-lamellar 30° (*p* < 0.0005). The differences in elastic modulus are not statistically significant between laminates pairs of +45°/–45° vs 45° and +60°/–60° vs 60° (Figure 5A). The UTS of 0°/90° laminate is 28% lower than that of 0° laminate (*p* < 0.005) but 3830% larger than that of 90° laminate (*p* < 0.0001) (Figure 5B). For compared laminate couples of +30°/–30° vs 30° and +45°/–45° vs 45°, for each couple, the UTS of the single lamella is significantly lower than the bilamella while showing a decrease of 44% (*p* < 0.05) and 57% (*p* < 0.05), respectively. However, the variation in UTS is lower for the +60°/–60° and 60° laminates (20% decrease). The ultimate strain of 0°/90° laminate is 8% less than 0° laminate but 41% more than 90° laminate (*p* < 0.0001) (Figure 5C). The +30°/–30° laminate demonstrates a 23% lower strain than the 30° laminate (*p* < 0.005), and the +45°/–45° and +60°/–60° laminates demonstrate much higher values compared with the 45° and 60° laminates, by showing 72% (*p* < 0.005) and 71% (*p* < 0.05) increase, respectively. The toughness of 0°/90° laminate is slightly smaller than 0° laminate while significantly larger than 90° laminate (*p* < 0.0005) (Figure 5D). Comparing combinations of similar angles, such as +30°/–30° vs 30°, +45°/–45° vs 45°, and +60°/–60° vs 60°, demonstrates that toughness values of bilamella are higher than single-lamella for each combination, with an increase of 61%, 222% (*p* < 0.005), and 110% (*p* < 0.05), respectively.

The geometric dimensions and properties (aspect ratio and FVF) of bilamellar biocomposites for morphing are detailed in supplementary Table S2. The properties are maintained relatively constant to allow the comparison of the shape transformation.

**Figure 6:**
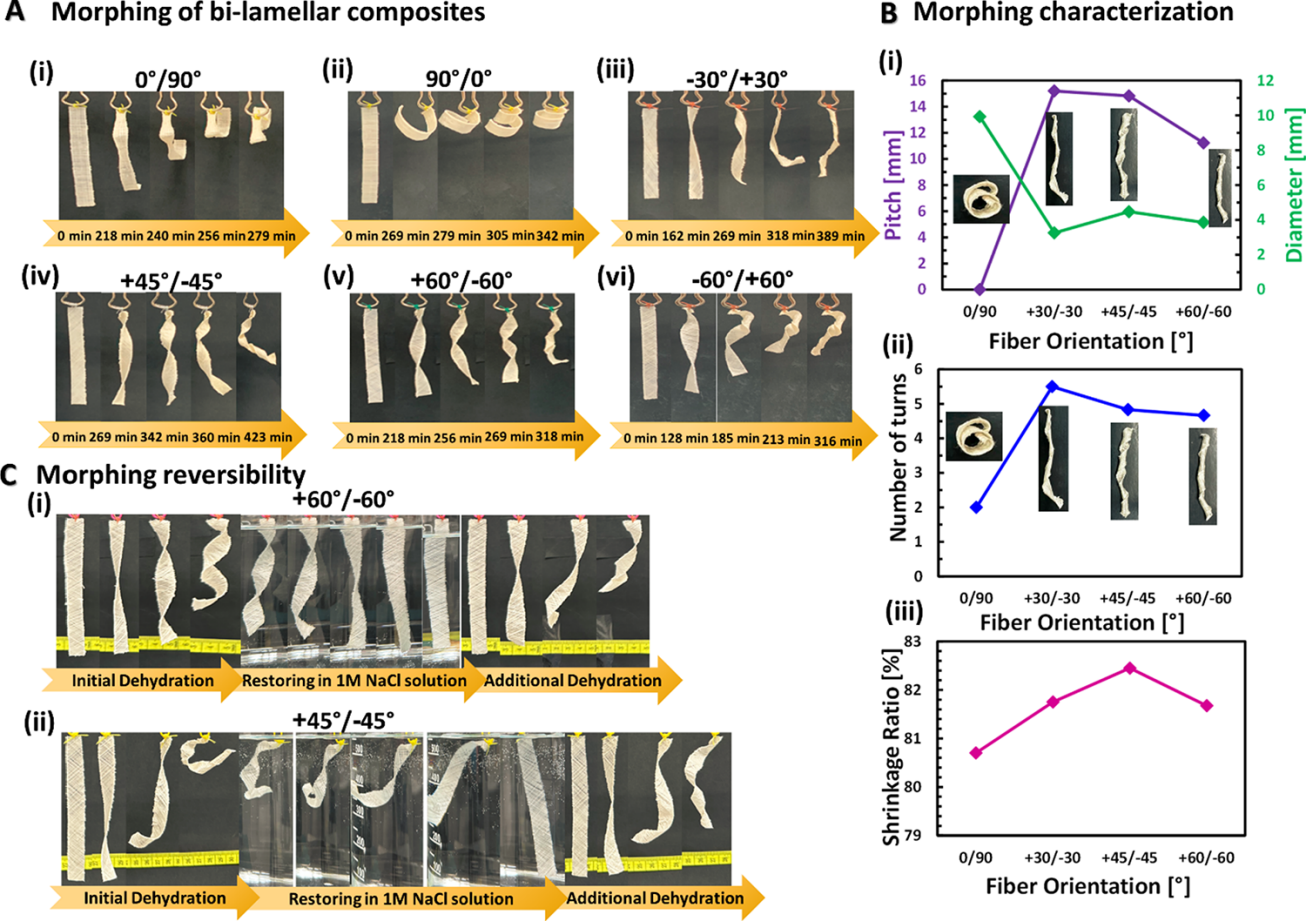
Morphing abilities, characterization, and reversibility. (A) Morphing of bilamellar biocomposites as a function of fiber orientation (i) 0°/90°, (ii) 90°/0°, (iii) −30°/+30°, (iv) +45°/–45°, (v) +60°/–60°, (vi) −60°/+60°. (B) Morphing characteristics of dried bilamellar biocomposites as a function of fiber orientation. (i) Pitch and diameter (*n* = 6), (ii) number of turns (*n* = 6), and (iii) shrinkage ratio (*n* = 5). (C) Morphing reversibility. Initial dehydration of the biocomposite until partially dry, then immersion in 1 M NaCl solution until restored to original shape. Finally, another dehydration until completely dry. (i) +60°/–60° and (ii) +45°/–45°.

As in plant tissues, our composites demonstrate multifunctionality. The planar strips change their simple 2D shape to a complex 3D configuration with chiral morphology, as seen in Figure 6A and supplementary Movie S1. The chirality shape evolution includes helical, coiled, and twisted forms, sometimes closed into cylindrical tubes, rolls, and rings. The hanging angle of the samples affects the handedness of the structural chirality. The final shape of cross-plied laminates, 0°/90° or 90°/0°, is a rolled ring backward-handed and forward-handed, respectively, as shown in Figure 6A(i–ii). For angle-plied laminates with various angle combinations, the achieved helical configuration closed into a tube (Figure 6A(iii,iv)). Upside-down hanging of identical angle-plied laminates, +60°/–60° and −60°/+60°, create an inverse handedness of left-handed and right-handed, respectively (Figure 6A(v,vi)). The width effect of bilamellar +45°/–45° composites is examined on the chiral configuration. Figure S2 (Supporting Information) shows that +45°/–45° laminates with larger widths obtain a helical form, whereas laminates with smaller widths achieve a twisted shape. In addition, the fully dried bilamellar laminates of 0°/90°, +30°/–30°, +45°/–45°, and +60°/–60° are characterized by the final shape as a function of the fiber orientation. As shown in Figure 6B(i), there is a drastic increase in pitch from 0 mm to 15.2 ± 2.1 mm between 0°/90° laminate and +30°/–30° laminate, respectively, and then a more moderate decrease to +45°/–45° laminate (pitch of 14.8 ± 3.7 mm) until +60°/–60° laminate (pitch of 11.2 ± 2.2 mm). The highest pitch value is obtained for the +30°/–30° laminate and the lowest for the 0°/90° laminate, for which there is no pitch at all. Conversely, there is a drastic decrease in the diameter between the 0°/90° and the +30°/–30° laminates from 9.9 ± 2.2 to 3.2 ± 0.2 mm, respectively. A diameter of 4.5 ± 0.6 mm is observed for the +45°/–45° laminate and 3.9 ± 0.6 mm for the +60°/–60° laminate. The maximum number of turns is obtained for the +30°/–30° laminate (5.5 ± 0.5 turns), while the minimum value of 2.0 ± 0.9 turns is achieved for the 0°/90° laminate (Figure 6B(ii)). The +45°/–45° and the +60°/–60° laminates have very similar values of the number of turns (4.8 ± 0.4 and 4.7 ± 0.5, respectively).

Figure 6B(iii) also demonstrates the trend of the shrinkage ratio of bilamellar biocomposites as a function of the fiber orientation. The highest shrinkage ratio of 82.4% is achieved by the +45°/–45° laminate, while the lowest ratio of 80.7% is obtained by the 0°/90° laminate. The +30°/–30° and +60°/–60° laminates demonstrate very similar shrinkage ratio values of 81.7%.

The reversibility of morphing is tested for +45°/–45° and +60°/–60° laminates in three stages of dehydration, hydration, and additional dehydration (Figure 6C). During the drying stage, the laminates shrink while changing shape from planar 2D to complex chiral 3D configuration. After the shape transformation, the laminates are dipped into a 1 M NaCl solution to rehydrate them, where they undergo swelling and return to their original flat shape. Then, the laminates are removed from the solution and again dried while undergoing 3D deformation (Supplementary Movie S2). The +45°/–45° laminate achieves a shrinkage ratio of 78% in the first dehydration process, a swelling ratio of 479% in the second hydration stage, and a final shrinkage ratio of 83% in the third stage. However, the +60°/–60° laminate demonstrates a 75% and 82% shrinkage ratio for the first and second dehydration processes, respectively, and a swelling ratio of 443% for the hydration stage.

**Figure 7:**
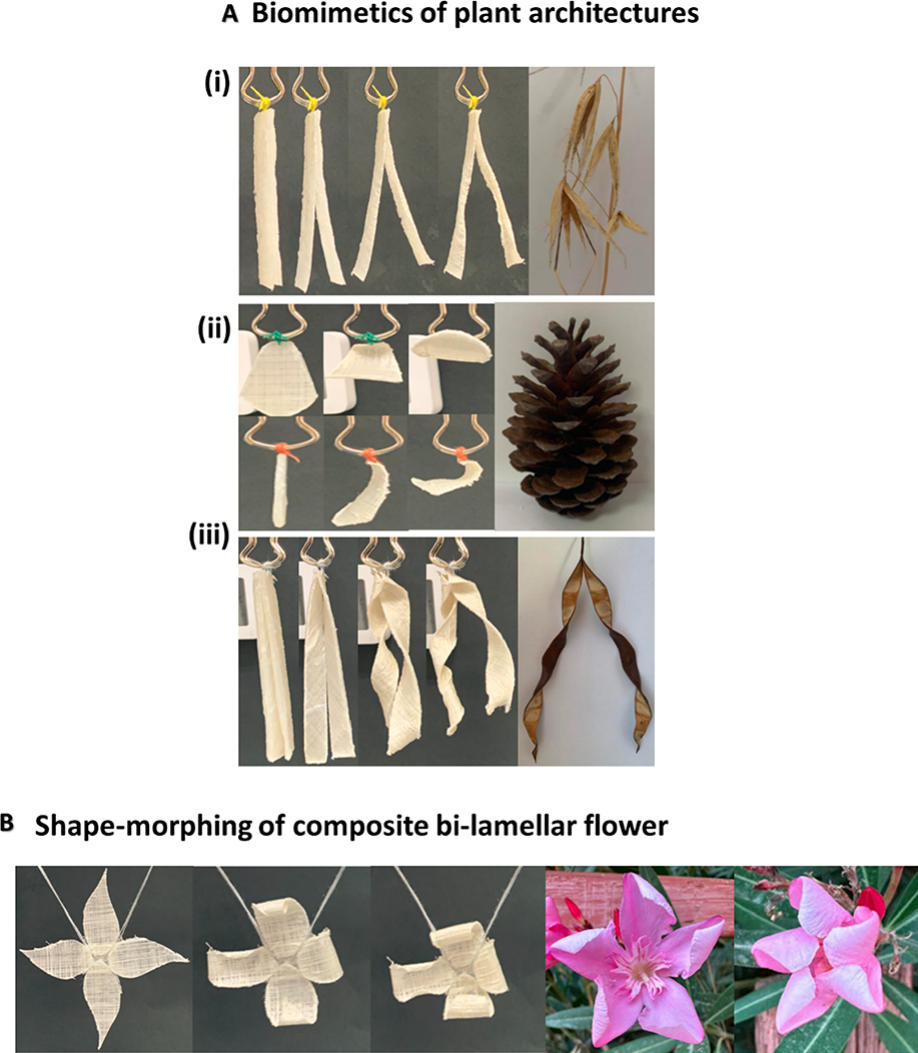
Biomimetic morphing abilities. (A) Biomimetics of morphing mechanisms of plants. (i) Wheat awn (0°/Random), (ii) pinecone scale (0°/90°), and (iii) seedpod (+45°/–45°). (B) Self-designed bilamellar complex flower structure with closing and folding ability (0°/90°) for every petal similar to the *Nerium oleander* flower.

The ability of the biocomposites to mimic the shape transformation behavior of various humidity-responsive plants using similar fiber orientations during their dehydration is presented in Figure 7A. The opening behavior of the wheat awn is similar to its opening ability in nature, as shown in Figure 7A(i) and seen in the supplementary Movie S3. The wheat spreads its two stalks apart from each other upon bending, and the pinecone demonstrated the bending ability of the scales in front and side views similar to nature (Figure 7A(ii) and supplementary Movie S4). The seedpod mimics its natural behavior by opening its two halves in opposite handedness, as shown in Figure 7A(iii). Each pod valve achieves a helically twisted structure (supplementary Movie S5).

A self-planned complex flower is fabricated and dried (Figure 7). The flower transformed its initial planar 2D shape to the final 3D programmed configuration. The complex architecture of the flower demonstrates a fold-closing behavior upon bending, as shown in Figure 7B and seen in supplementary Movie S6.

**Figure 8:**
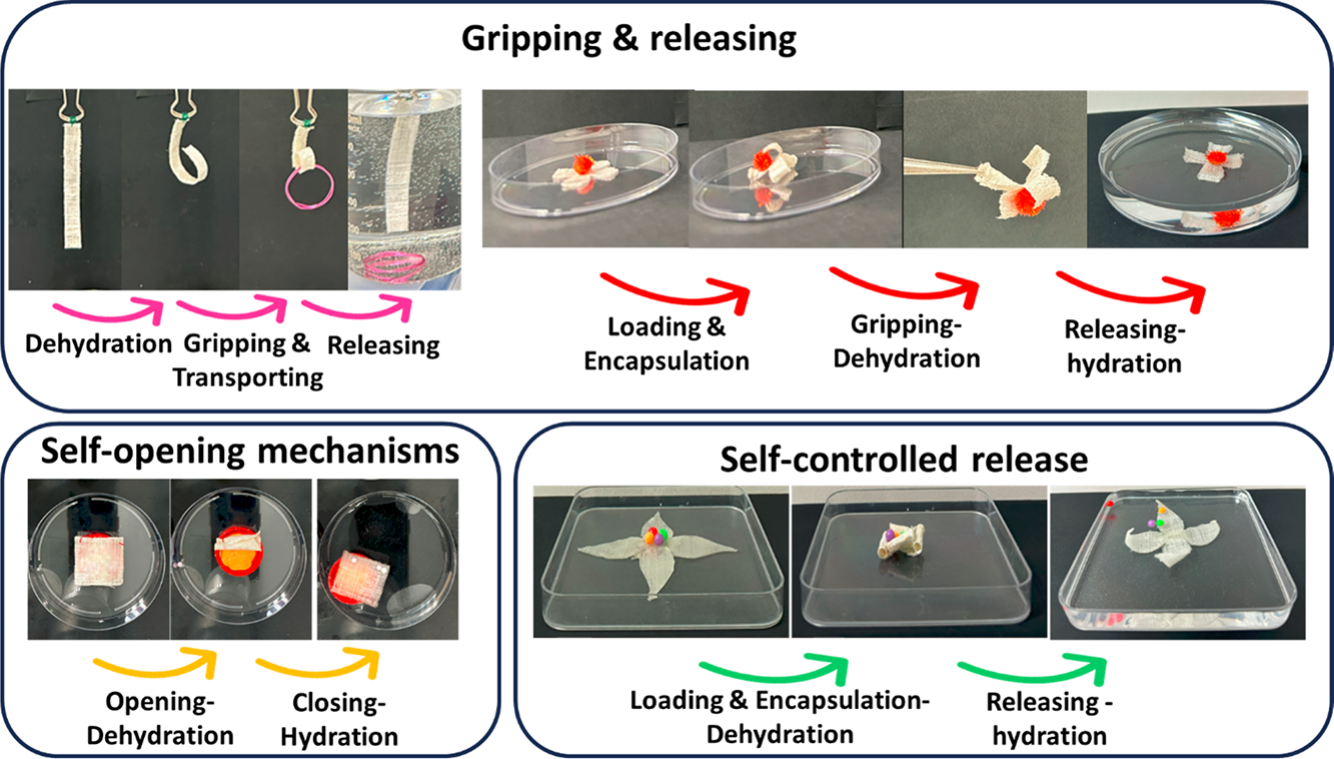
Proof-of-concept of shape-morphing applications, including gripping and releasing a metal ring, self-opening and closing membrane, and self-controlled release of small spheres.

We demonstrate the proof-of-concept of several applications, including gripping and releasing a metal ring, loading, encapsulating, and releasing small objects, and self-opening membrane (Figure 8 and Supplementary Movies S7–S10).

## 4 Discussion

Two systems of biocomposite laminates, single and bilamellar, were fabricated and structurally and mechanically characterized. These material systems were reinforced with different fiber orientations (0°, 30°, 45°, 60°, 90°). The single-lamellar laminate is constructed with a single layer of unidirectional fibers, whereas the bilamellar laminate is assembled from two layers with bidirectional fibers. These material systems are similar to the ones observed in plant tissues, where the interface between the stiff cellulose fibers and the softer hemicellulose matrix is governed by weak, reversible interactions, such as hydrogen bonds or van der Waals interactions. These interactions allow inner sliding as in other natural materials such as soft tissues.^11,26,33,37,38^ As in plant tissues, our laminates demonstrate stiff behavior in low fiber angles due to greater resistance to extension along their axis of alignment, whereas large fiber angles are considerably less stiff, and the bilamellar laminates present larger deformations.

Therefore, 0° laminate (longitudinal fibers) and 0°/90° laminate obtained the maximal stiffness, strength, and toughness, while the 90° laminate (transverse fibers) and +60°/–60° laminate achieved the minimal properties for single-lamellar and bilamellar laminates, respectively. The elastic modulus, UTS, and toughness showed a sharp and drastic decline between 0° and 30° laminates and a more moderate decrease until 90° laminate. This can be explained by the fact that the smaller the fiber angle with the stretching direction, the more the fibers can carry the load. A similar trend for the modulus and UTS, but not for toughness, was also shown for hard biological materials (wood and bone).^27^

The bilamellar laminates demonstrated greater mechanical properties for +30°/–30°, +45°/–45°, and +60°/–60° laminates compared to the same angles 30°, 45°, and 60° of single-lamellar laminates. The bilamella consisted of fibers aligned in the positive and negative directions relative to the tensile axis. Therefore, these laminates are more reinforced and can carry larger loads. The 0°/90° presented tensile properties in-between 0°- and 90°-oriented laminates since they consisted of a combination of longitudinal and transverse fibers, and the perpendicular fibers did not carry the load and tore (Figure 4B(v)). Therefore, they cause reduced properties compared to the 0°-oriented laminate. However, compared with the 0°-oriented single-lamellar composite, The 0°/90° laminate demonstrated smaller narrowing in the perpendicular direction.

These differences are allowed due to the enormous gap (several orders of magnitude) between the fiber and matrix stiffness.^11^ As in plant tissues, our laminates present nonlinear behavior and programmable shape-morphing ability in addition to diverse mechanical behaviors.

Furthermore, this coupling between the mechanical properties variance and morphing ability allows synergistic responses of cell walls where, for example, in pinecones, the cellulose fibers on the outer surface of the scale align to elongate when exposed to humidity, while the inner layer exhibits greater resistance to elongation.^26^

The silk fibers and alginate hydrogel matrix are both natural materials. Silk fibers are biological materials with impressive biocompatibility, environmental stability, flexibility, programmable and controllable biodegradability, and high mechanical properties of strength, stiffness, and toughness.^39−43^ Alginate is a biocompatible polysaccharide biopolymer with high viscosity that can react with polyvalent cations to form strong gels.^44,45^

The shape transformation was allowed due to the asymmetrical fiber arrangement of the biocomposite. Due to this mismatch, the coupling effect for in-plane/bending/twisting deformations was enabled.^12^ Additionally, the laminate matrix is composed of an alginate hydrogel with hydrophilic regions and cross-linked networks, which demonstrated absorbing and swelling/shrinking properties. During dehydration, the laminates are moisture-triggered, and their volumetric decreased by transferring water from the hydrogel networks and inducing shrinkage deformation.^28,46^ The combination of these two factors together allows the shape-changing of the material.

The bilamellar composites demonstrate shape-morphing during dehydration. Modifying the fiber orientation controls the final shape morphology from helical, spiral, and curling to tubes and rolls (Figure 6A). The hanging angle of the ribbons was significant for the resulting handedness of the obtained configuration. The chirality was equally formed for opposing hanging angles but at opposite handedness. There was a left-handed morphing for a positive hanging angle, while for a negative hanging angle, there was a right-handed morphing (Figure 6A(v,vi)). The aspect ratio is kept constant (0.1) for all morphing experiments (Supplementary Table S2). However, we also tested a smaller aspect ratio (0.07) (Supplementary Figure S2). When the width is larger, the laminate obtains a helical form, whereas laminates with smaller widths achieve a twisted shape, as also shown by Jeon et al.^27^

Inspired by plant architectures, similar transformations were achieved (Figure 7A), as shown also in previous studies.^1,3,8,25,47^ The movements obtained included bending for pinecone scales, twisting for seedpod valves, and opening the wheat awn stalks.

The representation of the bilamellar orientation of the different plants consists of simplification of the actual complex plant structure. For example, for the pinecone scale, we are based on the gross 0°/90° orientations based on the work of Studart and Erb,^1^ however, the actual orientations of the fibers are 30°/70°, as shown by Dawson et al.^48^

Additional self-planned complex flower structures demonstrated its foldability (Figure 7B). Its self-folding behavior gradually changed from an open state to a closed state (supplementary Movie S6). The silk-based composites achieved various shape-morphing modes such as curling, coiling, twisting, rolling, bending, and folding. These multifunctional materials have controllable morphing abilities. They can be used for diverse predesigned gripping and releasing applications, closing and opening membranes, and loading, encapsulation, and releasing, as seen in proof-of-concept designs in Figure 8 and supplementary Movies S7–S10).

Therefore, we demonstrate here material systems with large deformations and a wide range of moduli (3.0 ± 0.6–104.7 ± 11.3 MPa) and UTS (0.23 ± 0.04–12.5 ± 2.0 MPa) (Figure 5A,B) together with shape-morphing abilities. Therefore, we can propose to transfer principles from the plant world to the biomedical field. Our material systems can be combined and integrated with native soft tissues with similar mechanical behaviors, such as aorta (elastic modulus of 3–5 MPa and UTS of 0.3–1 MPa), skin (elastic modulus of 50–150 MPa and UTS of 1–30 MPa), articular cartilage (UTS of 9–40 MPa), and annulus fibrosus (elastic modulus of 31–77 MPa and UTS of 3.8–16 MPa).^38,49−51^

As in the plant world, this material system can be designed to actuate complex movements together with compatible mechanical support. Integrating these principles in the biomedical field can be further developed and assist research areas such as soft robotics, sensing, drug delivery, and biomedical engineering.

## 5 Conclusions

Our innovative biocomposite laminates demonstrated controllable 3D deformations and programmable mechanical properties. By using asymmetrical fiber alignment and the shrinkage of hydrogels, as in natural materials, shape transformation was achieved together with mechanical robustness. By tuning the fiber orientations, width dimensions, and hanging angle, we controlled the shape-morphing modes from coiling, twisting, bending, folding, and curling to cylindrical tubes and rolls. Furthermore, the laminates exhibited nonlinear behavior with large deformations and tunable stiffness, strength, and toughness as a function of the fiber orientation. Their biocompatibility and their mechanical anisotropic nonlinear behavior, showing large deformations similar to the behavior of natural soft tissues, make them ideal candidates for the integration with such tissues (e.g., aorta, skin, etc.), transferring from the plant world to biomedical applications thus opening new venues for biomedicine and bioengineering.

## Supporting Information Available

Orientation distribution of the biocomposites, width effect on final chiral morphology of +45°/–45° biocomposites, geometrical dimensions and mechanical properties of the tensile-tested biocomposite laminates, morphing characteristics of dried bilamellar biocomposites, shrinkage ratio of bilamellar biocomposites, and swelling/shrinkage properties of morphing reversibility (PDF)
Morphing in different fiber orientations (MP4)
Morphing reversibility of bilamellar +60°/–60° laminate (AVI)
Biomimetics of wheat awn behavior (AVI)
Biomimetics of pinecone scale bending (AVI)
Biomimetics of seedpod opening (AVI)
Self-designed complex structure of a large flower with morphing abilities (AVI)
Metal ring gripping and releasing (MP4)
Encapsulation and releasing (MP4)
Opening and closing membranes (MP4)
Loading, encapsulating, and releasing small spheres (MP4)

## Supplementary Material

ab3c01163_si_002.mp4

ab3c01163_si_003.avi

ab3c01163_si_004.avi

ab3c01163_si_005.avi

ab3c01163_si_006.avi

ab3c01163_si_007.avi

## Abbreviations

FVF: Fiber volume fraction
UTS: Ultimate tensile strength
