# Supplementary material for: Plant Biomimetic Principles of Multifunctional Soft Composite Development: A Synergistic Approach Enabling Shape Morphing and Mechanical Robustness
Source: ACS Biomater Sci Eng. 2024 Feb 21;10(6):3707–17. doi: 10.1021/acsbiomaterials.3c01163 (PMC11167591; doi:10.1021/acsbiomaterials.3c01163)
Supplement: Supplementary file 1 — ab3c01163_si_001.pdf [file ab3c01163_si_001.pdf]

Supporting Information

Plant Biomimetic Principles of Multifunctional Soft Composites Development: A Synergistic Approach Enabling Shape Morphing and Mechanical Robustness

Gital Shteinberg[1], Rami Haj-Ali[2], Flavia Libonati[3] and Mirit Sharabi [1,]*

[1] Department of Mechanical Engineering and Mechatronics, Ariel University, Ariel 407000, Israel

[2] School of Mechanical Engineering, Tel Aviv University, Tel Aviv 6997801, Israel

[3] Department of Mechanical, Energy, Management and Transportation Engineering, University of Genoa, Genova 16145, Italy

**Correspondence to:* Dr. Mirit Sharabi, Department of Mechanical Engineering and Mechatronics, Ariel University, Ariel 407000, Israel

Phone: +972-3-6453159

Email: miritsh@ariel.ac.il

Fiber orientation characterization

Figure S1 demonstrates the analysis of the orientation distribution and confirms the consistency and uniformity of the fiber orientation. A very narrow scatter and small variation around the desired angle are observed for all the tested bi-lamellar samples 0°/90°, +30°/-30°, +45°/-45°, and +60°/-60°.

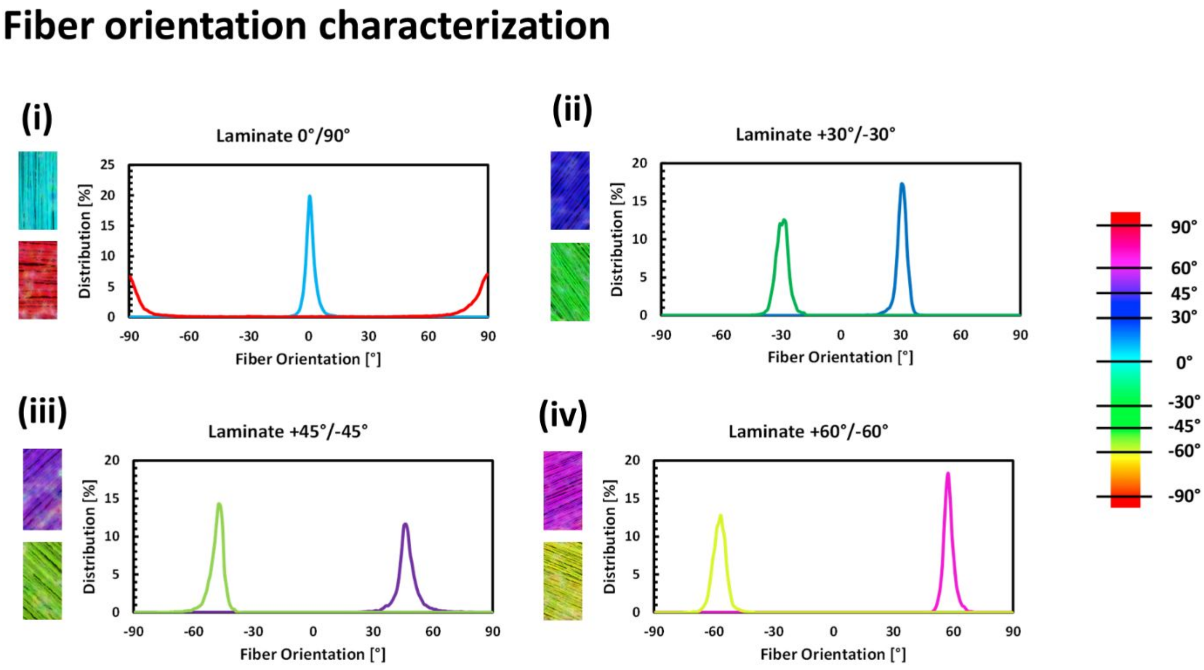

Figure S1: Fiber orientation of bi-lamella composites. **(i)** 0°/90°, **(ii)** +30°/-30°, **(iii)** +45°/-45°, **(iv)** +60°/-60°. The colormap spectrum represents the angle with the vertical axis.

The morphing of bi-lamella composites – width effect

Figure S2 presents the width impact on the final configuration of bi-lamella +45°/-45° laminates ribbons. The width effect is demonstrated by comparing a small width (6.5 mm) versus a large width (8.8 mm), when the strip length is constant.

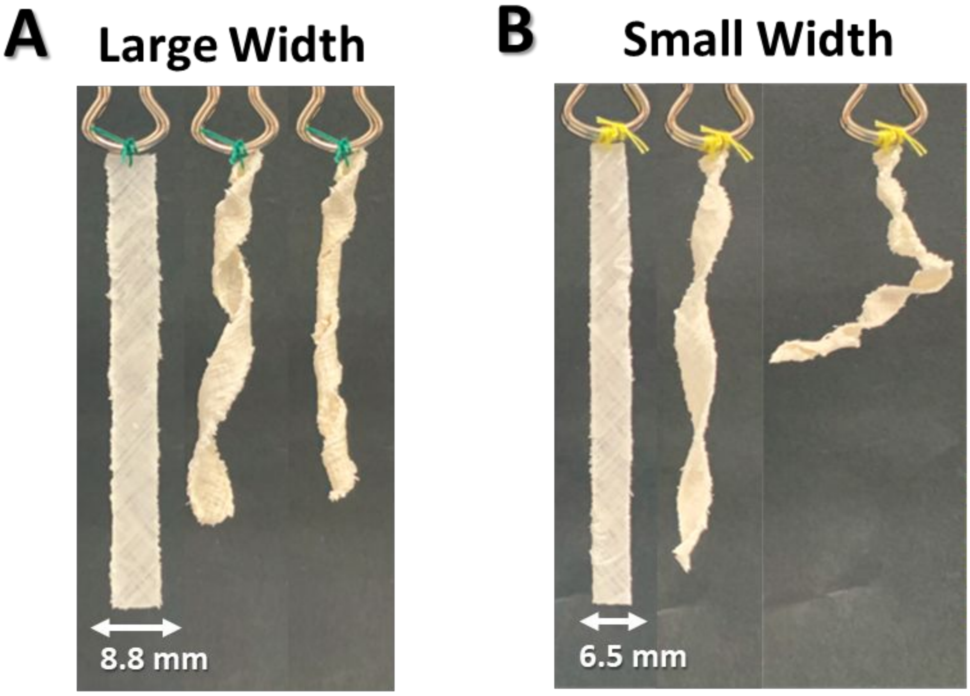

Figure S2: Width effect on final chiral morphology of +45°/-45° biocomposites. (A) Large width and **(B)** small width.

Supplementary Table S1: Geometrical dimensions and mechanical properties of the tensile-tested biocomposite laminates

Material System	n	Thickness [mm]	Width [mm]	Gage Length [mm]	Fiber Volume Fraction (FVF)	Aspect Ratio	Elastic Modulus [MPa]	UTS [MPa]	Ultimate Tensile Strain [mm/mm]	Toughness [MJ/m^3]
**0°**	6	0.83 ± 0.24	8.0 ± 0.3	15.8 ± 0.7	0.3 ± 0.09	0.51 ± 0.01	104.7 ± 11.3	12.5 ± 2.0	0.22 ± 0.02	3.5 ± 0.7
**30°**	6	0.84 ± 0.10	8.0 ± 0.2	15.7 ± 0.4	0.32 ± 0.05	0.51 ± 0.00	11.2 ± 0.7	1.4 ± 0.2	0.20 ± 0.02	0.23 ± 0.04
**45°**	6	0.79 ± 0.17	7.9 ± 0.3	15.8 ± 0.5	0.34 ± 0.04	0.50 ± 0.01	4.4 ± 0.7	0.37 ± 0.07	0.18 ± 0.03	0.07 ± 0.02
**60°**	6	0.78 ± 0.17	7.8 ± 0.3	15.4 ± 0.2	0.30 ± 0.04	0.50 ± 0.01	3.8 ± 1.4	0.31 ± 0.12	0.17 ± 0.07	0.06 ± 0.05
**90°**	6	0.75 ± 0.06	8.2 ± 0.4	16.2 ± 0.9	0.34 ± 0.02	0.51 ± 0.01	3.0 ± 0.6	0.23 ± 0.04	0.14 ± 0.01	0.03 ± 0.00
**0°/90°**	6	0.88 ± 0.15	8.3 ± 0.2	16.0 ± 0.3	0.67 ± 0.08	0.51 ± 0.01	79.9± 14.0	9.0 ± 1.7	0.20 ± 0.02	2.9 ± 1.0
**+30°/-30°**	5	0.95 ± 0.11	8.1 ± 0.2	16.1 ± 0.4	0.67 ± 0.05	0.50 ± 0.00	24.7 ± 3.5	2.5 ± 0.8	0.15 ± 0.02	0.36 ± 0.15
**+45°/-45°**	5	0.94 ± 0.06	8.1 ± 0.2	16.2 ± 0.6	0.59 ± 0.05	0.50 ± 0.00	4.9 ± 0.7	0.86 ± 0.25	0.32 ± 0.06	0.22 ± 0.08
**+60°/-60°**	5	0.84 ± 0.20	8.1 ± 0.2	16.2 ± 0.3	0.65 ± 0.12	0.50 ± 0.00	3.2 ± 0.4	0.38 ± 0.09	0.29 ± 0.06	0.13 ± 0.04

Supplementary Table S2: Geometric characteristics of the shape-transformed bi-lamella composite laminates

Material system	Thickness [mm]	Width [mm]	Length [mm]	Fiber Volume Fraction (FVF)	Aspect Ratio
**0°/90°**	1.61	9.9	90.6	0.35	0.11
**90°/0°**	2.24	9.9	90.4	0.40	0.11
**-30°/+30°**	2.03	9.9	90.3	0.37	0.11
**+45°/-45°**	2.59	9.7	90.7	0.40	0.11
**+60°/-60°**	1.95	9.4	90.4	0.38	0.10
**-60°/+60°**	1.55	9.9	90.3	0.42	0.11
**+45°/-45° (Large width)**	2.43	8.8	90.4	0.41	0.10
**+45°/-45° (Small width)**	1.86	6.5	90.6	0.52	0.07
